# Adult Granulosa Cell Tumor of the Testis: A Case Report with a Review of the Literature

**DOI:** 10.1155/2019/7156154

**Published:** 2019-05-19

**Authors:** Klaus-Peter Dieckmann, Julia Bertolini, Christian Wülfing

**Affiliations:** ^1^Asklepios Klinik Altona, Abteilung Urologie/Hodentumorzentrum, Hamburg, Germany; ^2^MVZ Hanse Histologikum, Hamburg, Germany

## Abstract

Adult granulosa cell tumor (AGCT) of the testis represents a very rare testicular neoplasm that is poorly understood clinically. Here we report the case of a 22-year-old male who presented with unspecific scrotal symptoms. Scrotal sonography disclosed a 6 mm hypoechoic intratesticular lesion. Histological examination after orchiectomy revealed a homogeneous and well demarcated neoplasm with monomorphic cells with nuclear grooving and microfollicular formation of the so-called Call-Exner bodies. Immunohistology showed positive stainings of vimentin, calretinin, and inhibin with negative stainings of the typical germ cell tumor markers. Thus, the diagnosis of a benign AGCT was made. The patient is well one year after surgery. A total of 91 previous AGCT cases were identified in the literature. Median age of the cases reported to date is 44 years, median tumor size 3.2 cm. 54.3% of the AGCT cases were located on the left side. 12 cases (13.2%) were of malignant nature. Testis-sparing surgery would be the treatment of choice, but only two of all cases had received that procedure. The present report aims to increase the clinical knowledge of AGCT and specifically to increase the clinician's vigilance with respect to testis-sparing surgery in probably benign testicular masses.

## 1. Introduction

Histologically, about 90% of all testicular neoplasms comprise germ cell tumors (GCTs) while the remainder involves sex cord gonadal stromal tumors, malignant lymphomas, secondary tumors, and other very rare new growths [1, 2]. Among the gonadal stromal tumors, Leydig cell tumors and Sertoli cell tumors are the most common subtypes encompassing 1-2% and 0.5%, respectively, of all testis tumors [3]. The third subtype is the granulosa cell tumor which represents a truly rare neoplasm. Granulosa cell tumors arising in the testis are histologically identical with their ovarian counterparts. With respect to the pathogenesis of these unusual testicular neoplasms, Teilum postulated that cells of the male gonadal stroma retain their embryological ability to differentiate into both male or female specialized stromal cells, i.e., Leydig or Sertoli cells and granulosa or theca cells, respectively [4]. Thus, in the case of neoplastic growth of gonadal stromal cells the tumor cell types of both genders may evolve. Regarding granulosa cell tumors, two subtypes must be considered, the juvenile type occurring only in the first year of life and the adult type occurring later in life [5]. The clinical behavior of gonadal stromal tumors and particularly that of the adult granulosa cell tumor (AGCT) is poorly understood so far. The overall experience rests on a few small case series [6] and predominantly on single case reports [7]. The aim of the present report is to document another case of AGCT and to summarize the knowledge regarding this rare tumor.

## 2. Case Presentation

**Figure 1:**
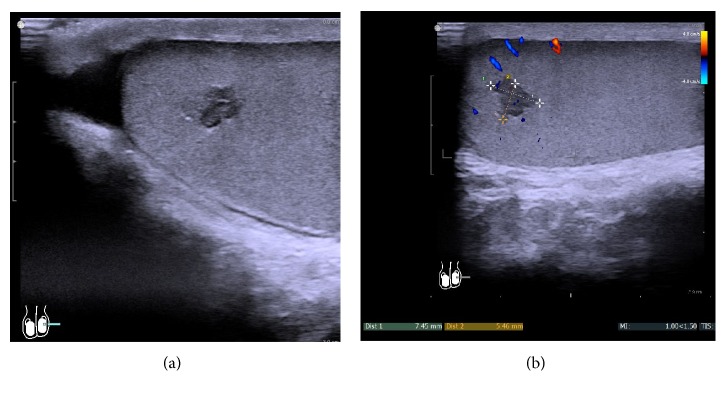
(a) Sonographic appearance of the adult granulosa cell tumor in the 22-year-old patient. Note the lobulated but well circumscribed shape and homogeneous hypoechoic appearance. (b) The same patient, color-coded duplex sonography showing duplex signals within the hypoechoic lesion.

This 22-year-old man of European descent presented with unspecific scrotal discomfort lasting for two weeks. Clinical history is uneventful. The patient is unmarried and has no children so far. He is an untrained workingman by profession. Both testicles were of normal size and nonsuspicious upon palpation with only some tenderness on the left scrotal side. Scrotal sonography revealed a 1 cm sized lobulated hypoechoic lesion at the cranial pole of the left testis (Figure 1(a)) showing intense color duplex signals within the lesion (Figure 1(b)). Laboratory workup disclosed no abnormal results. Specifically, the germ cell tumor markers alpha fetoprotein, beta-human chorionic gonadotropin, and lactate dehydrogenase were within normal limits, as were the serum testosterone level (6.57 ng/ml; reference limits 2.4–8.7) and the gonadotropins LH (1.69 U/l) and FSH (2.78 U/l). Upon surgical exploration, a well circumscribed greyish-tan lesion of about 1 cm in size was identified at the cranial pole. Inguinal radical orchiectomy with placement of a silicone testicular implant and contralateral testicular biopsy were performed accordingly. The postoperative course was uneventful. Computed tomography of chest and abdomen did not reveal any metastases. No further treatment was applied. The patient is well and without recurrence of the disease one year after surgery.

**Figure 2:**
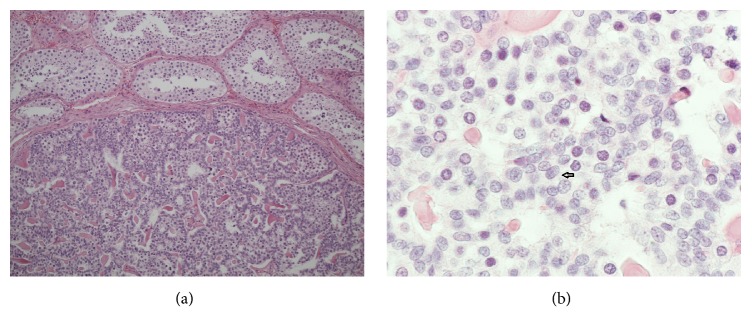
(a) Histological section of the neoplasm, hematoxylin-eosin stain. Well demarcated rim of the testicular neoplasm, no clear encapsulation. Normal testicular parenchyma, upper part of image. 10x magnification. (b) Histological section of the neoplasm. The tumor cells have scant cytoplasm and elongated nuclei with numerous grooves (arrow). Hematoxylin-eosin stain, magnification x20.

**Figure 3:**
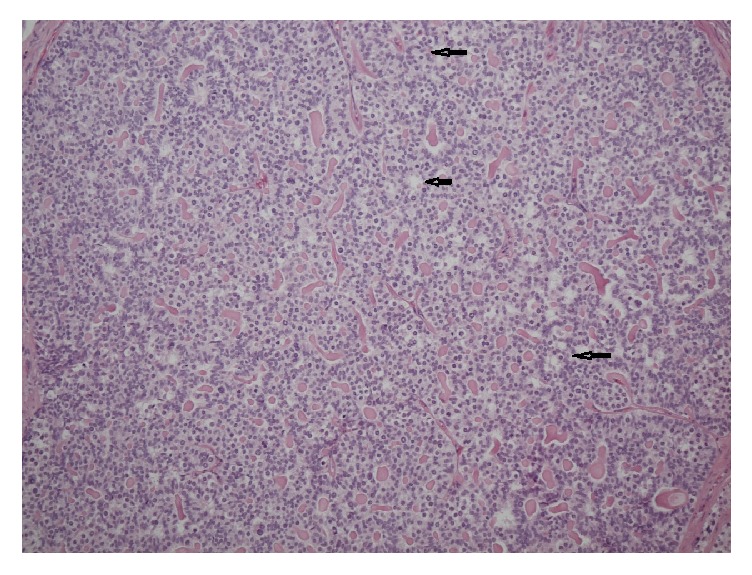
Histological section of the tumor. Adult granulosa cell tumor with microfollicular pattern. Call-Exner body: coffee bean-like tumor cells with groove core notches surrounding glandular cavities containing eosinophilic material and tumor cells detached from the dressing (arrows). Hematoxylin-eosin stain, 10x magnification.

**Figure 4:**
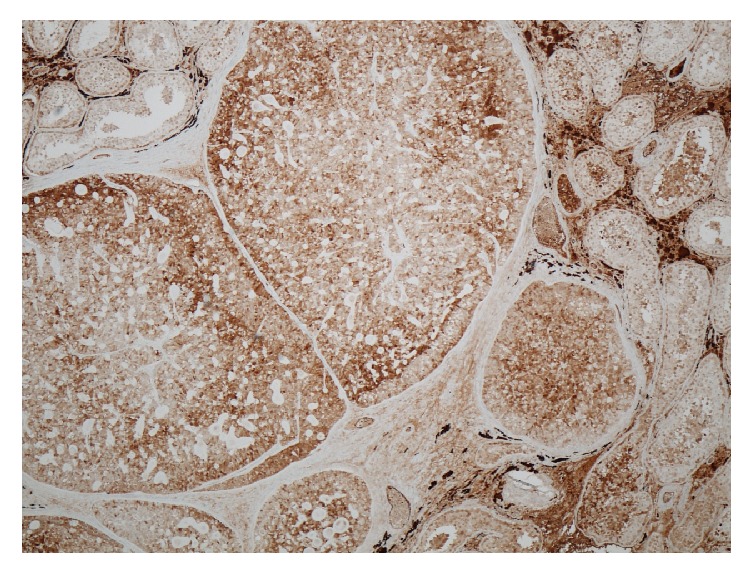
Immunohistological stain of calretinin. All neoplastic cells stain positive for calretinin, 10x magnification.

Pathohistological analysis showed a well demarcated solid neoplasm of 8 mm in size (Figure 2(a)). Histologically, the lesion consisted of monomorphic cells resembling typical ovarian granulosa cells. Many of the cells had large nuclei with many of them having a grooved shape mirroring the appearance of coffee beans (Figure 2(b)). The growth pattern was solid in most parts of the neoplasm but in some areas microfollicular structures prevailed resembling Call-Exner bodies (Figure 3). Mitotic figures were very rare. No necrotic areas were detected. The tumor-surrounding testicular tissue showed normal spermatogenesis. Germ cell neoplasia in situ was detected neither in the ipsilateral parenchyma nor in the contralateral testicular tissue. Immunohistochemical workup revealed positive staining of inhibin, vimentin, and calretinin (Figure 4) and negative staining for Oct 4 and chromogranin A. Staining of progesterone receptor was negative, too. The MIB-1 labeling index revealed a growth fraction of < 1%. The histopathological findings were thus consistent with the diagnosis of a benign adult type granulosa cell tumor of the testis.

## 3. Literature Survey

A total of 91 cases could be identified from the literature by using the PUBMED database and by hand-searching the reference lists of previous reports (Table 1). Not all of the case reports specified details regarding all of the clinical characteristics. Therefore, the various clinical features had to be analyzed with different sample sizes. With regard to age, 86 cases are eligible and the median age is 44 years (interquartile range (IQR) 26–55 years; range 12–87 years). With regard to tumor size, 80 cases are evaluable, with the median size amounting to 3.2 cm (IQR 1.5–5.0 cm; range 0.5–18.0 cm). Laterality is specified in 81 reports showing a slight preponderance of the left side with 44 cases (54.3 %; 95% confidence intervals (CIs) 42.9–65.4%) as opposed to 37 cases located on the right side (45.7%, 95% CI 34.6–57.1%). Malignancy was documented in 12 cases (13.2%) with 4 of these cases surviving more than 2 years. Endocrine symptoms were not systematically documented, but gynecomastia was reported in 8 cases. With regard to primary treatment, orchiectomy was the standard therapy. Only two cases had testis-sparing surgery.

## 4. Discussion

The first documentation of an AGCT is credited to Laskowski in 1952 [8]. By the year 2000, another 28 cases had been reported. In 2014, Schubert et al. summarized 43 cases of the literature [7]. In the same year, Cornejo et al. published the largest consecutive series of 32 patients [6]. Isolated cases not quoted by Schubert [36–39] and several cases documented since 2014 bring the total number to 91 cases reported to date (Table 1). Some more cases are briefly mentioned without providing details in several case series on gonadal stromal tumors [3, 55–57]. Thus, the total number of cases referred to in the medical literature is probably more than one hundred. And as a matter of fact, a large number of cases remained unreported. The number of documented cases has apparently increased since the year 2000. However, this statistical trend does probably not indicate a true increase of the incidence of AGCT but rather reflects a lower rate of misclassification of this rare entity because of increasing knowledge regarding testicular tumors [58]. One additional factor could be publication bias because the possibilities of publishing minor reports have much improved since the beginning of this century because of the ever increasing number of Internet-based scientific journals.

The age of the present patient is 22 years which is clearly lower than the median age of 44 years and even lower than the inter quartile range (26 -55 years) as observed in the literature survey. The lowest age ever observed in AGCT is 12 years [31] while the oldest subject is aged 87 years [6].

The tumor size of 8 mm found in our case is clearly smaller than the median size of 3.2 cm observed in the literature survey and it lies in the lowest quartile of reported findings. Obviously, granulosa cell tumors of the adult type may occur with a wide variety of sizes since seven cases with tumor sizes of less than 1 cm were reported (Table 1) and on the other side there were at least eight patients with giant tumors sized >10 cm. The median size of AGCT is very similar to the sizes of 3.2–3.5 cm and 2.8–3.5 cm found in nonseminomatous and seminomatous germ cell tumors, respectively [59, 60]. Probably, the presenting size of different histological types of testicular tumors does not relate to biological reasons but rather to sensual-cognitive structures of patients. Putatively, a diameter of around 3 cm represents the threshold where the majority of men will recognize a mass in the testicle. Most of the patients with AGCT presented with slowly growing painless testicular masses with several of whom reporting a duration of symptoms for many years [7, 22, 30]. On the other hand, eight cases were sized <1cm that were found incidentally upon autopsy [9] or with ultrasonography [25, 43]. Overall, a palpable mass is probably the typical presenting symptom of AGCT as it is with testicular germ cell tumors [6, 38, 45]. Endocrine-related symptoms were infrequently reported in previous cases, but eight patients of the survey were reported to present with gynecomastia. This symptom is not specific for AGCT because it is frequently observed in Leydig cell tumors and sometimes also in beta-human chorionic gonadotropin secreting germ cell tumors [61]. Our patient presented with unspecific scrotal symptoms that were not related to the tiny testicular tumor that was only detected with ultrasonography. Sonographic features were frequently reported and typically involve hypoechoic well circumscribed masses with positive color-coded sonographic signals [37, 49]. But anechoic areas corresponding to cystic parts of the tumor have also been observed [7, 52].

Regarding laterality of AGCT, our patient presented with a left sided tumor which corresponds to the findings in the majority of cases of the survey where 54.3% (95% CI 42.9–65.4%) had left sided tumors as opposed to 45.7% (95% CI 34.6–57.1%) right sided tumors. However, in view of the overall small database and overlapping confidence intervals of the proportions there is probably no clear evidence for a predisposition of one side. The same is true with germ cell tumors where no clear preponderance of one particular side has been found [59].

The neoplasm detected in our patient was a clearly benign lesion. Among the 91 cases listed in the survey, 12 were malignant (13.2%). A 15% proportion of malignancy was likewise reported by Rove et al. [57] and two additional malignant cases were briefly mentioned by Featherstone et al. [56]. Reportedly, clinical features of malignancy involve tumor size > 5 cm and age > 50 years [34, 57]. Histologically, >3 mitoses per high-power field, invasion of rete testis, vascular invasion, and cellular atypia are considered as characteristics of malignancy [51, 58].

Histologically, AGCT consist of monomorphic cells identical with ovarian granulosa cells with a scanty cytoplasm. Many of the cells may show coffee bean-like nuclei with small grooves [6, 45]. The cells are mostly arranged in trabeculae and sheets and some may form microfollicular structures, the so-called Call-Exner bodies [62]. The histological diagnosis mainly rests on immunohistochemical findings with positive stainings of inhibin, vimentin, and calretinin and negative stainings for pancytokeratin and the typical germ cell tumor specific markers like placental alkaline phosphatase, D2-40, and Oct 3/4 [7, 18, 20, 21, 62].

With regard to treatment, testis-sparing surgery would be the treatment of choice, as only few cases are malignant. As age >50 years and tumor size are the most important characteristics of malignancy the cases qualifying for conservative surgery could in principle be easily identified, preoperatively.

Hence, only two of the cases of the survey received that appropriate therapy and also the present case was submitted to orchiectomy. Clearly, in everyday clinical practice it is difficult to sort out benign tumors from malignant ones by means of clinical assessment only. As malignant tumors of the testis are far more common than benign growths most of the urologic surgeons will proceed to radical orchiectomy. However, as shown in the present case and in many cases of the survey, benign tumors of the testis do actually occur, and small size is one of the leading characteristics of benign nature though not specific. The present case report is thought to increase the overall knowledge of testicular granulosa cell tumors, and it particularly aims to increase the vigilance of urologic surgeons to consider conservative surgery in cases with small testicular neoplasms. Practically, a conservative approach with intraoperative frozen section examination could help to save more testicles and avoid unnecessary orchiectomies.

## Figures and Tables

**Table 1:** Synopsis of cases with adult granulosa cell tumor of the testis reported in 1953–2018.

*Author* (first)	*year*	*reference *(#)	*age* (years)	*tumor size* (cm)	*right side*	*malignant*	*presenting symptom*	*endocrine symptoms*	*F/U*
Laskowski	1952	[8]	35	9,00	R			gynecomastia	8,5 yrs
Cohen	1953	[9]	21	<1 cm	L		incidental	gynecomastia	ns
Massachusetts case *∗*	1955	[7]	53	10 cm	R			gynecomastia	ns
Melicow	1955	[10]	52	13,00	R				ns
Mostofi	1959	[11]	41	10,10	L	malignant		gynecomastia	DOD 5 m
Marshall	1977	[12]	53	10,00	ns		mass, no pain	gynecomastia	17 yrs
Talerman	1985	[13]	44	3,50	R		mass, no pain		3 yrs
Gaylis	1989	[14]	41	1.8 cm	R				ns
Düe	1990	[15]	83	18 cm	L		mass, no pain		DOC 1 m
Nistal	1992	[16]	61	5,00	R				ns
Matoska	1992	[17]	26	10,00	L	malignant	painful mass	gynecomastia	14 yrs NED
Sasano	1992	[18]	ns	ns	ns				ns
Monobe	1992	[19]	42	ns	L	malignant			AWD
Jiminez-Quintero	1993	[20]	57	2,50	R				3y DOC
Jiminez-Quintero	1993		55	1,30	L				ns
Jiminez-Quintero	1993		60	7,00	L	malignant			DOD 11 yrs
Jiminez-Quintero	1993		39	4,00	L				3 yrs NED
Jiminez-Quintero	1993		29	7,50	R	malignant			
Jiminez-Quintero	1993		76	0,70	L				NED 1 m
Renshaw	1997	[21]	ns	ns	ns				ns
Renshaw	1997		ns	ns	ns				ns
Renshaw	1997		ns	ns	ns				ns
Morgan	1999	[22]	49	2,60	L				2 yrs NED
Al-Bozom	2000	[23]	48	5,00	R				7 m NED
Wang	2002	[24]	54	ns	L		hydrocele, asymptomatic		ns
Guzzo	2004	[25]	33	1,00	L				ns
Suppiah	2005	[26]	51	ns	L	malignant			6 yrs AWD
Hisano	2006	[27]	59	15,00	L				4 yrs NED
Arzola	2006	[28]	32	2,10	L		painless mass		
Lopez	2007	[29]	77	4,00	L				ns
Ditonno	2007	[30]	45	6,50	R		painless mass		2 yrs NED
Gupta	2008	[31]	12	8,00	L				1 yr NED
Hammerich	2008	[32]	55	ns	ns	malignant			3 yrs NED
Song	2011	[33]	28	2,60	L				ns
Hanson	2011	[34]	21	1,00	L				2 yrs NED
Lima	2012	[35]	77	2,50	R				ns
Lima	2012		22	1,00	L				ns
Lima	2012		40	2,10	L				
Schubert	2014	[7]	78	1,3	L		painless mass		23 m NED
Cornejo	2014	[6]	49	3,00	R				137 m, DOOD
Cornejo	2014		22	3,30	L				48 m
Cornejo	2014		50	0,90	L				59 m
Cornejo	2014		ns	3,00	L				lost
Cornejo	2014		27	1,80	R				60 m
Cornejo	2014		87	ns	ns				169 m
Cornejo	2014		47	4,50	R				lost
Cornejo	2014		51	2,10	R				58 m
Cornejo	2014		47	0,50	L				lost
Cornejo	2014		55	2,10	L				80 m
Cornejo	2014		25	2,00	R				lost
Cornejo	2014		45	1,40	R				lost
Cornejo	2014		74	3,50	L				104 m
Cornejo	2014		27	1,40	R				36 m
Cornejo	2014		32	4,20	R				lost
Cornejo	2014		18	3,20	L				lost
Cornejo	2014		60	6,00	R				lost
Cornejo	2014		23	1,40	R				65 m
Cornejo	2014		20	4,50	L				1 m
Cornejo	2014		52	4,30	R	malignant			24 m AWD
Cornejo	2014		14	0,80	R				lost
Cornejo	2014		21	3,00	R				37 m
Cornejo	2014		17	1,50	R				lost
Cornejo	2014		64	2,40	R				28 m
Cornejo	2014		14	3,50	R				35 m
Cornejo	2014		28	2,60	L				lost
Cornejo	2014		51	3,00	L				lost
Cornejo	2014		63	1,00	L				25 m
Cornejo	2014		25	5,50	R				1 m
Cornejo	2014		44	1,20	L				1 m
Cornejo	2014		50	4,70	R				1 m
Köksal	2003	[36]	40	3,50	R		8-month mass	no	20 m
Mitra	2008	[37]	25	0,50	L		scrotal pain 2 weeks		ns
Kucukodaci	2008	[38]	21	1,50	L		incidental	no	1 yr
Harrison	2009	[39]	65	5,00	L	malignant			DOD
Miliaris	2013	[40]	37	4,20	L		painless mass	no	2 yrs
Norman	2013	[41]	68	ns	R				18 m
Rane	2014	[42]	34	5,00	R				6 m
Rane	2014		46	65,00	L				2,5 yrs
Rane	2014		46	11,00	R	malignant			3 m
Tanner	2014	[43]	22	0,60	L				ns
Tsitouridis	2014	[44]	29	2,50	R				1 yr
Giulianelli	2015	[45]	80	6,00	R		mass, no pain	gynecomastia	1 yr
Vallonthaiel	2015	[46]	43	6,00	L		painless mass		1 yr
Gomez-Valcarcel	2016	[47]	23	ns	ns				ns
Mohapatra	2016	[48]	57	4,7	L	malignant	incidental	gynecomastia	32 m NED
Al-Alao	2016	[49]	48	1,20	L				ns
Bani	2016	[50]	20	2,00	R				4 m NED
Elbachiri	2017	[51]	40	5,50	L	malignant	painless growing mass		2 yrs NED
Mezentsev	2017	[52]	74	5,00	R		painless mass		ns
Meilan	2017	[53]	59	3,30	ns				ns
Nunes-Carneiro	2017	[54]	31	4,50	ns				10 yrs

*∗*As cited by Schubert et al. 2014.

Ns: not stated.

NED: no evidence of disease; DOD: dead of disease; DOC: dead of complication; AWD: alive with disease; DOOD: dead of other disease.

yrs: years; m: months.

R: right side; L: left side; F/U: follow-up.
